# *mbtD* and *celA1* association with ethambutol resistance in *Mycobacterium tuberculosis*: A multiomics analysis

**DOI:** 10.3389/fcimb.2022.959911

**Published:** 2022-08-31

**Authors:** Zhuhua Wu, Qiuchan Tan, Chenchen Zhang, Yuchuan Zhao, Qinghua Liao, Meiling Yu, Liuyue Xu, Jiawen Wang, Hongdi Liang, Haicheng Li, Liang Chen, Xunxun Chen, Wenjing Wei

**Affiliations:** ^1^ Key Laboratory of Translational Medicine of Guangdong, Center for Tuberculosis Control of Guangdong Province, Guangzhou, China; ^2^ School of Basic Medical Sciences, Guangzhou Health Science College, Guangzhou, China; ^3^ The Third Affiliated Hospital, Sun Yat-Sen University, Guangzhou, China

**Keywords:** ethambutol, Mycobacterium tuberculosis, resistance-related genes, drug resistance, multi-omics

## Abstract

Ethambutol (EMB) is a first-line antituberculosis drug currently being used clinically to treat tuberculosis. Mutations in the embCAB operon are responsible for EMB resistance. However, the discrepancies between genotypic and phenotypic EMB resistance have attracted much attention. We induced EMB resistance in *Mycobacterium tuberculosis in vitro* and used an integrated genome–methylome–transcriptome–proteome approach to study the microevolutionary mechanism of EMB resistance. We identified 509 aberrantly methylated genes (313 hypermethylated genes and 196 hypomethylated genes). Moreover, some hypermethylated and hypomethylated genes were identified using RNA-seq profiling. Correlation analysis revealed that the differential methylation of genes was negatively correlated with transcription levels in EMB-resistant strains. Additionally, two hypermethylated candidate genes (*mbtD* and *celA1*) were screened by iTRAQ-based quantitative proteomics analysis, verified by qPCR, and corresponded with DNA methylation differences. This is the first report that identifies EMB resistance-related genes in laboratory-induced mono-EMB-resistant *M. tuberculosis* using multi-omics profiling. Understanding the epigenetic features associated with EMB resistance may provide new insights into the underlying molecular mechanisms.

## Introduction

Pre-coronavirus pandemic, tuberculosis (TB) was the main cause of death from a single infectious agent and is one of the most common communicable diseases worldwide (Kirby, 2021). According to the WHO Global TB Report of 2021, approximately 1.5 million people died from TB in 2020 (Chakaya et al., 2021). Drug-resistant TB (DR-TB) is a major global public health problem. Globally, approximately 3%–4% of new and 18%–21% of previously treated TB cases return with multidrug resistance (MDR) or rifampicin (RFP) resistance (RR) (Seung et al., 2015). Drug resistance in TB complicates the effectiveness of anti-TB drugs (Lee and Suh, 2016). The mechanism of *Mycobacterium tuberculosis* (Mtb) drug resistance remains unknown, and new anti-tuberculosis drugs are scarce.

Ethambutol (EMB) is an arabinose analogue that interferes with arabinosyl transferase, resulting in the obstruction of cell wall synthesis in mycobacteria. This makes it a key drug in first-line anti-TB treatment (Zhang et al., 2020). A national survey of drug-resistant TB indicated that the resistance rate to EMB has gradually increased in China, approaching 17.2% in re-treated TB patients (Zhao et al., 2012). Some studies revealed that mutations in the embCAB operon play a major role in the development of EMB resistance in Mtb, especially the “canonical” mutations in codon 306, 406, or 497 of embB (Telenti et al., 1997; Plinke et al., 2010; Safi et al., 2010; Safi et al., 2013). In addition, mutations in *aftA* (Safi et al., 2013), *embR* (Sharma et al., 2006), and *ubiA*, which are involved in the biosynthesis of the mycobacterial cell wall (He et al., 2015), are also associated with variable levels of EMB resistance in Mtb. Despite a strong association between gene mutation and EMB resistance, mutations in genes cause variable degrees of EMB resistance and are required, but not enough, to cause high-level resistance to EMB (Palomino and Martin, 2014). Approximately 30% of EMB-resistant strains do not present any mutations in embB (Palomino and Martin, 2014), and many clinical strains present with mutations in this gene but remain susceptible to EMB (Safi et al., 2013).

Over the past decade, epigenetic mechanisms have become increasingly important in our understanding of pathogenicity, host immunity, hypoxic survival, and virulence. Epigenetics can affect gene expression without altering the DNA sequence, not only in eukaryotes but also in bacteria, in which DNA methylation is one of the main epigenetic mechanisms for the regulation of gene expression (Fatima et al., 2021). Studies on the correlation between DNA methylation and mechanisms of drug resistance in Mtb have recently attracted extensive attention. The role of DNA methylation in gene regulation and stress response in Mtb has been identified as a novel mechanism by which Mtb modulates gene expression in the stress response (Shell et al., 2013). Single-molecule, real-time (SMRT) sequencing profiled the core methylome of clinical isolates and provided a comprehensive list of methylated genes in drug-resistant clinical isolates (Gong et al., 2021). Furthermore, DNA methylation has become a new direction to study TB drug resistance, but there are no reports exploring its relationship with EMB-resistant Mtb.

In this study, we induced mono-EMB-resistant Mtb *in vitro* and used an integrated genome–methylome–transcriptome–proteome approach to study the microevolutionary mechanism of EMB resistance. Two genes, *mbtD* and *celA1*, were further investigated and were found to be associated with EMB resistance. We hope that this study can help reveal a new Mtb EMB resistance mechanism and provide a new target and basis for reversing multidrug resistance.

## Materials and methods

### Bacterial strains and growth conditions

The wild-type Mtb H37Rv strain was used as the parent strain. A total of 12 clinical Mtb isolates, consisting of six drug-susceptible and six mono-EMB-resistant Mtb strains, were obtained from sputum samples of patients with pulmonary TB who presented to the Centre for Tuberculosis Control of Guangdong Province, Guangzhou, Guangdong, China. All isolates were tested using both the MYCOTB and the Löwenstein–Jensen (LJ) proportion method against 14 anti-TB drugs. Mtb strains were cultured on LJ, Middlebrook 7H9, and 7H10 media with OADC (oleic acid–albumin–dextrose–catalase). The cultures were grown at 37°C.

### *In vitro* induction of EMB-resistant Mtb

Before laboratory evolution, the Mtb H37Rv strain was cultivated in LJ medium without the drugs used for laboratory evolution. A monoclone was picked and processed by amplification culture and used as the primary strain (ERG0), which was selected to prepare the EMB-resistant strain. ERG0 was cultivated on LJ medium containing a series of concentration gradients (2^−4^, 2^−3^, 2^−2^, 2^−1^, and 2^0^) of EMB, based on the World Health Organization (WHO) critical concentration (2.0 mg/L EMB) (World Health Organization, 2018). ERG0 cells were cultured on LJ medium (2^−4^ EMB concentration) for approximately 4 weeks and named generation 1 (ERG1). These steps were repeated until the culture met the WHO criteria for the EMB-resistant strain (ERG7). To stabilize the drug-resistant phenotype, the ERG7 EMB-resistant Mtb strain was continuously cultured until generation 8 (ERG8) for further analysis. The wild-type Mtb H37Rv strain, simultaneously cultivated on LJ medium without EMB, was used as a control strain (WTG8). All generation strains were verified using the appropriate concentrations of the drug used in the drug-susceptibility test. Each generation’s cells were stored in glycerol stocks at −80°C for further analysis.

### Drug susceptibility testing

The Mtb isolates were subjected to susceptibility testing against anti-tuberculosis drugs using the standard proportion method on LJ medium according to previously described procedures (Canetti et al., 1969). The Sensititre MYCOTB MIC plate (BASO) was used to determine the minimum inhibitory concentrations (MICs) of Mtb strains according to the manufacturer’s instructions. Each 96-well microtiter plate was used to test an isolate against 14 anti-tuberculosis drugs: isoniazid (INH), streptomycin (SM), ethambutol (EMB), ofloxacin (OFX), moxifloxacin (MFX), amikacin (AKM), kanamycin (KM), capreomycin (CPM), protionamide (PTO), *p*-aminosalicylate (PAS), rifampin (RFP), rifabutin (RBU), levofloxacin (LFX), and pyrazinamide (PZA). Visible cell growth in a drug-free well indicated usable results. The MIC was recorded as the lowest antibiotic concentration that reduced visible growth. Three independent experiments were performed.

### Whole-genome resequencing

Mtb genomic DNA was extracted and purified using traditional cetyltrimethylammonium bromide according to previously published procedures (Somerville et al., 2005). Whole-genome resequencing and bioinformatics analyses were performed as previously described (Zhao et al., 2019). Library preparation and genome sequencing were briefly performed using Gene-Optimal (Shanghai, CA). A TruSeq DNA kit (Illumina) was used for DNA library preparation. The libraries were sequenced using Illumina MiSeq or HiSeq 2000 sequencing systems. High-quality reads were mapped to the H37Rv reference genome (NC_000962.3) using the Geneious 6.0 (Biomatter).

### SMRT sequencing

Genomic DNA was extracted from the ERG8 and WTG8 strains. SMRT library preparation from genomic DNA samples, SMRT sequencing (Pacific Biosciences RSII platform), and bioinformatics analysis were performed as previously described (Zhu et al., 2016).

### Methylation-specific PCR

Methylation-specific PCR (MSP) was performed as previously described (Ku et al., 2011). DNA extracted from the ERG8 and WTG8 strains were subjected to sodium bisulfite treatment and DNA purification using the EZ DNA Methylation-Gold Kit (Zymo Research), according to the manufacturer’s instructions. MethPrimer (http://www.urogene.org/methprimer/), an online platform, was used for primer design (Li and Dahiya, 2002). The MSP primers used in this study are listed in **Supplementary Table S1**. We used the universal methylated human DNA standard (Zymo Research) as a fully methylated (100%) MSP positive control. A set of two PCR reactions were performed, and the products were analyzed *via* gel electrophoresis.

### RNA-seq analysis

Total RNA was isolated from Mtb strains using FastPrep-24 with a FastRNA Pro Blue Kit (MP Biomedicals) following the manufacturer’s instructions. Total RNA fragmentation was performed using the ultrasonic method (140–160 bp) (Covaris M220) after DNase I (QIAGEN) treatment and ribosomal RNA removal (Epicentre). Random primers were used to synthesize the first and second strands, and dTTP was replaced with dUTP for complementary (cDNA) synthesis. Ultra-™Directional RNA Library Prep Kit for Illumina (NEB) was used according to the manufacturer’s instructions. The final library products were purified using 0.8× beads (Beckman) and assessed using an Agilent Bioanalyzer 2100 system (Agilent). The Illumina HiSeq 4000 platform was used for the whole-transcriptome analysis.

### Reverse transcription quantitative PCR

Total RNA was extracted and converted to cDNA using a SuperScript III First-Strand Synthesis Kit (Thermo Fisher Scientific). The mRNA expression of three genes (*mbtD*, *mbtB*, and *celA1*) was analyzed using SYBR Green (Maeda et al., 2003). SigA was used as the internal reference. Mean ± SEM was calculated from three independent experiments, and significant differences were determined using the unpaired Student’s *t*-test. The primers used in this study are listed in **Supplementary Table S1**.

### Protein preparation and liquid chromatography–mass spectrometry analysis

Mtb total protein was extracted by the mechanical crushing method, digested with trypsin (37°C for 24 h), and labeled using the iTRAQ(R) Reagents Multiplex Kit (Sigma) following the manufacturer’s instructions. The samples were subjected to liquid chromatography–mass spectrometry (LC–MS) according to our published procedure (Wu et al., 2020). Three biological replicates were used for each group.

### Bioinformatics analysis

Pairwise comparison of the average methylation levels across the samples by the median methylation levels. The heatmap was created using the HemI 1.0 software (|fold change| > 2, *p*_adj_ < 0.05). Gene ontology (GO) enrichment and KEGG analyses were performed using DAVID online software.

### Statistical analyses

Data are presented as mean ± standard deviation (SD) for each group. Student’s *t*-tests were used to compare the differences between two independent groups. The correlation between differential methylation and gene expression levels was determined using Spearman’s linear regression analysis. Statistical significance was set at *p* < 0.05.

## Results

### Laboratory microevolution of Mtb under EMB stress conditions

**Figure 1:**
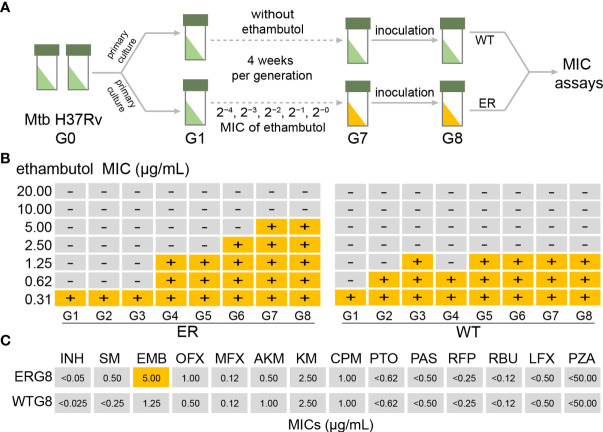
Laboratory evolution of *Mycobacterium tuberculosis* (Mtb) under ethambutol (EMB) stress conditions. **(A)** Schematic overview of laboratory evolution. Mtb strains at each generation were stored in glycerol at −80°C and subjected to minimum inhibitory concentration (MIC) measurements. **(B)** Time series of MIC value of 14 anti-TB drugs for each generation during the laboratory microevolutionary experiment. Using Mtb H37Rv as a wild-type strain, the laboratory microevolutionary experiments were repeated for seven generations until the MIC reached the critical concentration. **(C)** The MIC values of 14 anti-TB drugs in ERG8 strain. Critical concentrations for drug susceptibility testing by Löwenstein–Jensen (LJ) medium, 2.0 μg/ml. Critical concentrations for DST by liquid culture, 5.0 μg/ml.

**Figure 2:**
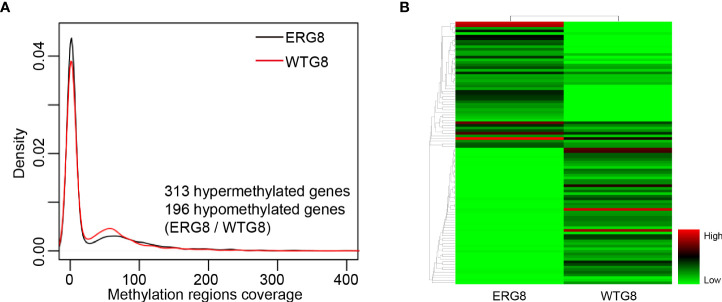
DNA methylation profiling. **(A)** Probability density of the DNA methylation region coverage by strain. The black line indicates the respective ERG8 strain. The red line indicates the WTG8 strain, **(B)** Heatmap showing the hierarchal clustering of the differential methylated genes. Each column represents an individual sample, and each row represents a gene. Methylation levels are depicted according to the color legend on the right. Color change from green to red indicates the change from hypomethylation to hypermethylation.

To globally investigate EMB-resistant mechanism of Mtb, we performed a laboratory microevolutionary strategy of Mtb using EMB stimulation with successive concentration gradients. **Figure 1A** shows a schematic overview of the laboratory evolution protocol. **Figure 2B** shows the time course of MIC change during laboratory evolution. To analyze molecular mechanisms associated with resistance acquisition, we expected to obtain a mono-EMB-resistant Mtb strain. Therefore, the MIC values of 14 anti-TB drugs were determined in 96-well microplates. The results indicated that the MICs of all anti-TB drugs except EMB remained below the WHO criteria under continuous EMB drug pressure; that is, no cross-resistance occurred, indicating that the induction of EMB-resistant Mtb was successful (**Figure 1C** and **Supplementary Table S2**).

### Whole-genome resequencing of the induced EMB-resistant Mtb

To identify mutations that confer resistance against EMB, we performed whole-genome sequencing analysis of induced EMB-resistant Mtb. Compared with the parental strain, comparative genomic analysis revealed that apart from the point mutation within *embB* (1489C→A, G497L) of EMB-resistant strains (ERG8 and WTG8), a point mutation occurred in *ubiA* (113C→T, A38V). These results indicate that EMB-resistant Mtb was successfully induced and established. Interestingly, a point mutation at C1489A in the *embB* gene was found in four EMB-susceptible isolates (ERG3, ERG4, ERG5, and ERG6). However, no mutations were found in the ubiA gene of EMB-susceptible isolates (ERG1–ERG6). No other drug-resistance related gene mutations were found in EMB-resistant strains (**Figure 1B**, **Supplementary Table S3**). These results suggest that in addition to these two gene mutations, there may be other factors influencing EMB resistance in Mtb.

### Association of aberrant methylation with gene expression

**Figure 3:**
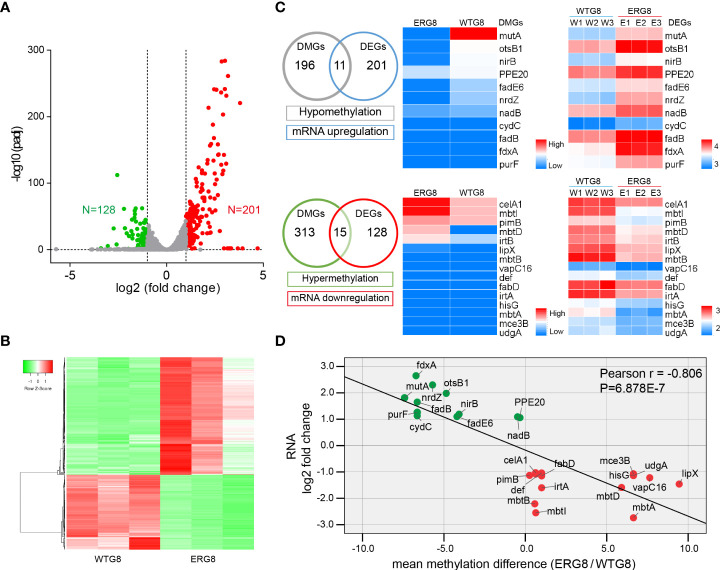
Association analysis of aberrant methylation with gene expression. **(A)** Volcano plot showing differentially expressed genes. Red, upregulated genes, green, downregulated genes. Gray, genes that are not significantly differentially expressed. *p*_adj_, adjusted *p*-value. **(B)** Hierarchical clustering of the 329 differentially expressed genes (|fold change|>2, *p*_adj_ < 0.05). **(C)** Overlap between DNA methylation and RNA expression changes. Left panel, Venn diagram showing the numbers of consistent DNA methylation-gene expression linkage events. Significantly high/low levels of 11/15 genes’ expression and low/high levels of methylation were observed. Middle panel, heatmap clustering of the screened differentially methylated genes (DMGs); the gradient of blue to red color represents an increase of the median methylation level. Right panel, a heatmap of the relative abundance of the screened differentially expressed genes (DEGs). Colors denote log 10 relative abundance of each gene in each sample. The relative abundance is shown in red (high), white (middle), and blue (low). **(D)** The correlation between gene expression levels and DNA methylation status. The green dots represent the 11 identified low-methylation and high expression genes. The red dots represent the 15 identified high-methylation and low expression genes.

Potential aberrant methylation of genes in the ERG8 strain was identified with the SMRT sequencing assay identifying the genome-wide distribution of methylated sites in ERG8 and WTG8. Compared with the WTG8 strain, 509 aberrant methylation of genes were found, including 313 hypermethylated genes and 196 hypomethylated genes in the ERG8 strain (**Figures 2A, B**, **Supplementary Table S4**). DNA methylation is linked to gene silencing, which is considered a key mechanism in the regulation of mRNA transcription. We subsequently investigated the association between aberrant methylation and gene expression in the ERG8 strain by transcriptome analysis. We identified 3,982 genes in total, including 201 that were significantly upregulated and 128 that were significantly downregulated (*p*_adj_ ≤ 0.05, |log 2 Ratio|≥1) (**Figures 3A, B**, **Supplementary Table S5**).

Consistent DNA methylation–gene expression linkage events were extracted by dividing the differentially methylated genes (DMGs) and differentially expressed genes (DEGs) into four expression groups based on their methylation/expression levels (hypermethylation, hypomethylation, mRNA upregulation, and mRNA downregulation). Clustering analysis revealed that the high expression group showed a low methylation level (11 genes), whereas the low-expression group showed a high methylation level (15 genes) (**Figure 3C**). In addition, correlation analysis revealed that differential methylation of genes was negatively correlated with transcription levels in the EMB-resistant strain (**Figure 3D**).

### Identification of differentially expressed proteins in the induced EMB-resistant Mtb

**Figure 4:**
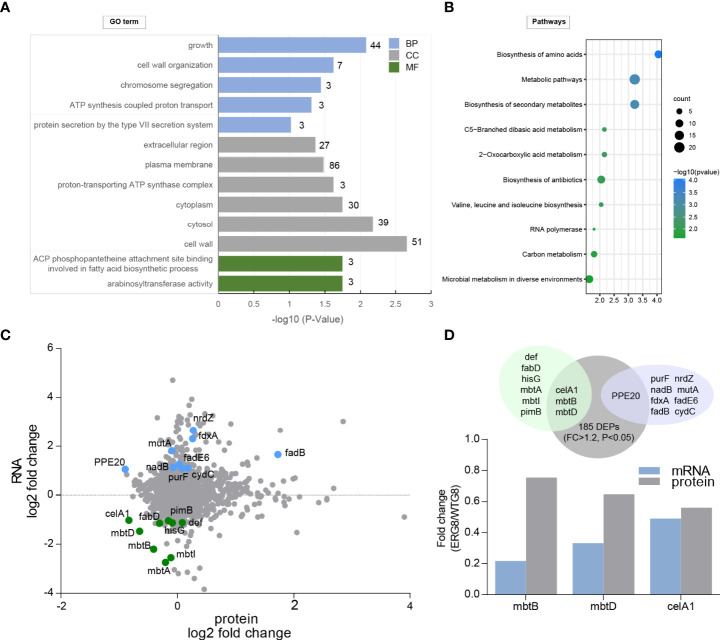
Identification of differentially expressed proteins (DEPs) in laboratory-induced strains. **(A)** Gene ontology (GO) enrichment analysis of the identified differentially expressed protein based on cellular component (CC), molecular function (MF), and biological processes (BP). **(B)** KEGG pathway enrichment analysis. **(C)** Integration of proteome and transcriptome datasets. **(D)** Overlap between protein and RNA expression changes. Top panel, proteins (gray) were mapped to protein-coding transcripts. Downregulated transcripts (light green) and upregulated transcripts (light purple) were assessed for overlap. Bottom panel, significant and consistent changes at protein and RNA.

Changes in protein levels and expression of genes in the laboratory-induced EMB-resistant strain were investigated. We performed global protein expression profiling by iTRAQ-two-dimensional LC–MS/MS on the ERG8 and WTG8 strains. A total of 2,175 proteins were identified, and 185 proteins had expression differences greater than 1.2-fold (*p* < 0.05), including 100 upregulated proteins and 85 downregulated proteins in the ERG8 strain compared to the WTG8 strain (**Supplementary Table S6**). GO enrichment and KEGG pathway analyses were also performed to classify the functions of significantly expressed proteins. As a result, 60 differentially expressed proteins (DEPs) were associated with growth, 86 and 51 DEPs were related to cellular components of the plasma membrane and cell wall, respectively, and DEPs mainly participated in the arabinosyltransferase activity and ACP phosphopantetheine attachment site binding involved in the fatty acid biosynthetic process (*p* < 0.05) (**Figure 4A**). KEGG pathway analysis showed that these proteins were primarily involved in biosynthesis and metabolic pathways (**Figure 4B**). We subsequently investigated the association of DEGs between transcriptional and protein expression levels by integrating transcriptomic and proteomic analyses. Of the 11 identified low-methylation and high-expression genes, 9 genes were identified at the protein level according to the proteomic dataset. Accordingly, 15 genes with high methylation and low expression were identified, and 9 genes were identified at the protein level according to the proteomic dataset, among which 3 genes (*mbtB*, *mbtD*, and *celA1*) were significantly downregulated (**Figure 4C**). The changes (EMG8 vs. WTG8) in the candidate genes at the transcriptional and protein levels are presented in **Figure 4D**.

### Experimental validation of differential genes screened by multi-omics in EMB-resistant Mtb

**Figure 5:**
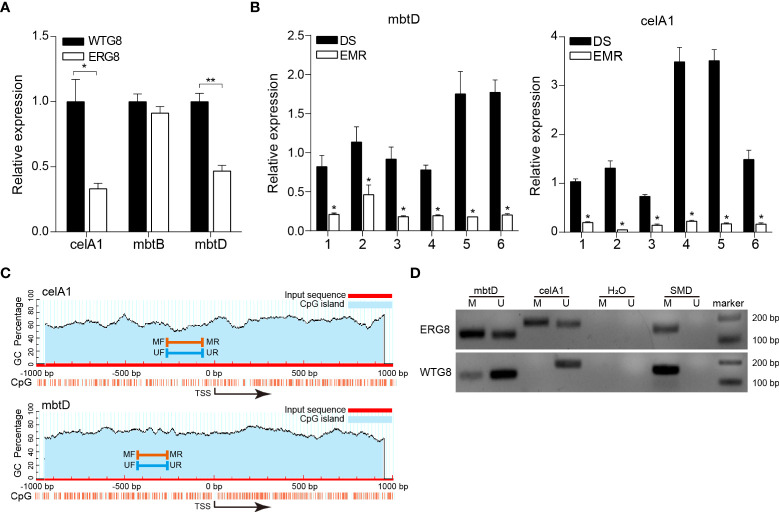
Validation of *mbtD* and *celA1* expression and promoter methylation status. **(A)** Expression of *mbtB*, *mbtD*, and *celA1* in ERG8 and WTG8 strains was tested by quantitative PCR. **(B)** Quantitative PCR was used to further verify the expression of *mbtD* and *celA1* in drug susceptibility (DS) (*n* = 6) and mono-EMB (ethambutol)-resistant (EMR) (*n* = 6) strains. For each gene, the left bar represents the DS cohort, and the right bar represents the EMR cohort. *p < 0.05 for EMR vs DS. DS, drug-susceptible clinical isolates of *Mtb*. EMR, mono-EMB-resistant clinical isolates of *Mtb*. **(C)** Schematic diagrams of CpG islands in the promoter region of *mbtD* and *celA1*. MF, methylation forward primer. MR, methylation reverse primer. UF, unmethylation forward primer. UR, unmethylation reverse primer. **(D)** Methylation status of *mbtD* and *celA1* was detected by MSP in ERG8 and WTG8 strain. SMD, positive controls. M, methylated alleles. U, unmethylated alleles. ** *p* < 0.01

DNA methylation, especially within the promoter regions, is traditionally correlated with gene expression. To further validate the association between aberrant methylation and gene expression, three genes, *mbtD*, *mbtB*, and *celA1*, were selected to detect methylation status and mRNA expression levels by MSP and qPCR; qPCR confirmed that mRNA expression levels of *mbtD* (*p* < 0.01) and *celA1* (*p* < 0.05) were significantly higher in the WTG8 strain than in the ERG8 strain. The mRNA expression of *mbtB* was not significantly different between the ERG8 and WTG8 strains (*p* > 0.05) (**Figure 5A**). In addition, we analyzed mRNA expression levels of *mbtD* and *celA1* in 12 confirmed clinical isolates of Mtb, namely, six drug-susceptible (DS) and six mono-EMB-resistant (EMR) species; the MICs of 14 anti-TB drugs are shown in **Supplementary Table S7**. The expression of *mbtD* and *celA* was significantly downregulated in the EMR strains compared to that in the DS strains (**Figure 5B**). CpG islands are situated in the *mbtD* and *celA1* gene promoter regions and the designed MSP primers are shown in **Figure 5C**. MSP was used to evaluate the methylation status. Partial ethylation of *mbtD* and *celA1* was observed in the ERG8 strain, and unmethylation of *mbtD* was observed in the WTG8 strain. In contrast, partial methylation was detected in the WTG8 strain with low expression of *celA1* (**Figure 5D**).

## Discussion

In the present study, we conducted laboratory microevolution of mono-EMB-resistant Mtb *in vitro* and performed multi-omics analysis of laboratory-induced EMB-resistant Mtb using an integrative methylome–transcriptome–proteome assay. Genome-wide DNA methylation and RNA-seq analyses identified patterns of differential DNA methylation and mRNA expression in Mtb. By integrating proteomic data, two candidate genes (*mbtD* and *celA1*) were screened and verified by qPCR, which corresponded to changes in DNA methylation.

In contrast to the method of agar-based selection for laboratory evolution against antibiotics (Maeda et al., 2021) or induced by high-concentration drugs (Zhao et al., 2014), the strategy of a continuous drug concentration gradient can better simulate the accumulation of drugs in the human body. Determination of the MIC value of 14 anti-TB drugs showed that laboratory-induced mono-EMB drug-resistant strains were successfully obtained (**Supplementary Table S2**). EMB resistance development is mainly caused by mutations in related genes, and according to recent literature, mutations in *embCAB*, *embR*, and *ubiA* genes are responsible for approximately 70% of clinical EMB-resistant Mtb (Xiang et al., 2021). Despite the strong association between genetic mutations and EMB resistance, related gene mutations have also been found in clinical EMB-susceptible strains (Mokrousov et al., 2002). The most common *embCAB* mutations in EMB-susceptible strains have been previously described in Iran, with phenotypic susceptibility to EMB reconfirmed (Khosravi et al., 2019). In our study, we performed whole-genome sequence analysis of induced EMB-resistant/susceptible Mtb. The most common mutation, Gln497Lys, in the *embB* gene of EMB-susceptible isolates located was noticed. Phenotypic drug susceptibility testing was repeated for all laboratory-induced EMB-resistant/susceptible Mtb strains, and preliminary results were confirmed. Accordingly, we believe that additional mechanisms of EMB resistance other than mutations in these sequences within the *embCAB* operon should also be considered.

DNA methylation is an important regulatory mechanism leading to differential gene expression, and *m5C* and *m6A* methylation have been found in Mtb genomes (Zhu et al., 2016). Additionally, it regulated expression of genes involved in Mtb hypoxic survival (Shell et al., 2013). A comprehensive list of methylated genes in drug-resistant clinical isolates that shows how DNA methylation genes are involved in drug resistance of Mtb is available (Gong et al., 2021), suggesting that gene methylation could be the focus of research on drug-resistant TB. However, the extent and functional consequences of DNA methylation in bacteria are poorly studied. We first characterized the methylomes of a laboratory-induced mono-EMB-resistant strain using SMRT sequencing. A total of 509 methylated genes were identified. We found that many genes progressively acquired or lost DNA methylation owing to the development of EMB resistance.

We subsequently investigated the association between aberrant methylation of genes and gene expression in laboratory-induced mono-EMB-resistant strain. Consistent with published articles (Brenet et al., 2011; Blattler et al., 2014; Anastasiadi et al., 2018), in this study, we observed that hypermethylated genes are negatively correlated with their transcriptional levels. The relative expression levels of 15 genes (*celA1, mbtI, pimB, mbtD, irtB, lipX, mbtB, vapC16, def, fabD, irtA, hisG, mbtA, mce3B*, and *udgA*) were decreased based on transcriptome analysis; conversely, the hypermethylation of these genes was observed in mono-EMB-resistant strain. Similarly, 11 genes (*mutA, otsB1, nirB, PPE20, fadE6, nrdZ, nadB, cydC, fadB, fdxA*, and *purF*) expression levels substantially increased based on transcriptome analysis, and hypomethylation of these genes was observed in mono-EMB-resistant strains. We identified three genes (*mbtD*, *mbtB*, and *celA1*) that were hypermethylated and downregulated transcriptionally as well as at the protein level. MSP is a useful tool for qualitative DNA methylation analysis (Huang et al., 2013). For the validation of gene methylation status, we specifically chose traditional MSP for the analysis of promoter hypermethylation. In summary, we demonstrated that *mbtD* and *celA1* are hypermethylated and downregulated in mono-EMB-resistant strains. To our surprise, the mRNA expression of mbtB was not significantly different between the ERG8 and WTG8 strains (*p* > 0.05). A cluster of 10 genes (designated mbtA–J), including mbtB, mbtE, and mbtF, form an assembly line of nonribosomal peptide synthetases and polyketide synthases (mbtC and mbtD) that activate and elongate the monomers of the mycobactin core (LaMarca et al., 2004; Nde et al., 2011). It has been shown that IdeR acts as a transcriptional repressor that directly affects the transcription of the mbtB (Gold et al., 2001); however, no differential expression of IdeR was observed between the ERG8 and WTG8 strains (**Supplementary Table S5**). Furthermore, deletion of the *mbtB* gene results in the limited growth of *M. tuberculosis* H37Rv in iron-limited media, but normal growth in iron-replete media (De Voss et al., 2000). *celA1* belongs to glycosyl hydrolase family 6 with cellulase function (Vaca-Gonzalez et al., 2020). Cellulose is an important component of the biofilm matrix and is involved in biofilm formation (Limoli et al., 2015). When overexpressed in *M. smegmatis* or *M. bovis* BCG, *celA1* and its homologue, *MSMEG_6752* or *BCG0063* gene, could impede biofilm production (Van Wyk et al., 2017). Numerous studies have reported the function of mycobacterial biofilms and have shown increased antimicrobial tolerance of biofilms compared to planktonic cells (Ojha et al., 2008; Islam et al., 2012). Chakraborty et al. demonstrated that the administration of nebulized cellulase enhanced the antimycobacterial activity of isoniazid and rifampicin in infected mice, supporting the role of biofilms in phenotypic drug tolerance (Chakraborty et al., 2021). Savijoki et al. (2021) also reported that increased *celA1* synthesis in *M. marinum* prevents biofilm formation and leads to reduced rifampicin tolerance. These results suggest that c*elA1* expression is linked to biofilm formation and antibiotic tolerance. Our data demonstrate that mono-EMB-resistant strains show dysregulation of *mbtD* and *celA1*, both at the level of methylation and gene expression, which could be an underlying mechanism behind the development of EMB resistance. However, whether overexpression of *mbtD* and *celA1* modulates susceptibility to EMB in the setting of EMB resistance remains to be demonstrated. Therefore, in our future studies, *mbtD* and *celA1* will be our targets for further exploration of EMB-resistant Mtb.

In conclusion, the integrated analysis of the methylome–transcriptome–proteome has provided a resource for genes whose expression, at least partially, is regulated by DNA methylation and may be involved in the development of EMB resistance in Mtb. We demonstrated that *mbtD* and *celA1* were hypermethylated and downregulated in a mono-EMB-resistant strain. These results provide a better understanding of the mechanisms involved in EMB resistance development.

## Data availability statement

The datasets presented in this study can be found in online repositories. The names of the repository/repositories and accession number(s) can be found in the article/**Supplementary Material**.

## Ethics statement

Ethical review and approval was not required for the study of human participants in accordance with the local legislation and institutional requirements. Written informed consent from the patients/participants was not required to participate in this study in accordance with the national legislation and the institutional requirements.

## Author contributions

ZW, WW, XC, and LC conceived, designed, and supervised the study. ZW, QT, and WW acquired funding and supervised and administered the project. ZW, CZ, YZ, and LX processed samples and performed experiments. ZW, QL, MY, JW, HCL, and HDL analyzed the data. ZW and QT wrote the manuscript. All authors contributed to the article and approved the submitted version.

## Funding

This work was supported by the Youth Innovative Talents Project in Colleges and Universities in Guangdong Province (grant number 2021KQNCX208), the Science and Technology Projects in Guangzhou (grant number 202201011764), the Major Infectious Disease Prevention and Control of the National Science and Technique Major Project (grant number 2018ZX10715004), the Medical Scientific Research Foundation of Guangdong Province (grant number A2020611), and the Guangdong Provincial Natural Science Foundation (grant number 2020A1515010658).

## Conflict of interest

The authors declare that the research was conducted in the absence of any commercial or financial relationships that could be construed as a potential conflict of interest.

## Publisher’s note

All claims expressed in this article are solely those of the authors and do not necessarily represent those of their affiliated organizations, or those of the publisher, the editors and the reviewers. Any product that may be evaluated in this article, or claim that may be made by its manufacturer, is not guaranteed or endorsed by the publisher.
